# GXD: a community resource of mouse Gene Expression Data

**DOI:** 10.1007/s00335-015-9563-1

**Published:** 2015-05-05

**Authors:** Constance M. Smith, Jacqueline H. Finger, Terry F. Hayamizu, Ingeborg J. McCright, Jingxia Xu, Janan T. Eppig, James A. Kadin, Joel E. Richardson, Martin Ringwald

**Affiliations:** The Jackson Laboratory, Bar Harbor, ME 04609 USA

## Abstract

The Gene Expression Database (GXD) is an extensive, easily searchable, and freely available database of mouse gene expression information (www.informatics.jax.org/expression.shtml). GXD was developed to foster progress toward understanding the molecular basis of human development and disease. GXD contains information about when and where genes are expressed in different tissues in the mouse, especially during the embryonic period. GXD collects different types of expression data from wild-type and mutant mice, including RNA in situ hybridization, immunohistochemistry, RT-PCR, and northern and western blot results. The GXD curators read the scientific literature and enter the expression data from those papers into the database. GXD also acquires expression data directly from researchers, including groups doing large-scale expression studies. GXD currently contains nearly 1.5 million expression results for over 13,900 genes. In addition, it has over 265,000 images of expression data, allowing users to retrieve the primary data and interpret it themselves. By being an integral part of the larger Mouse Genome Informatics (MGI) resource, GXD’s expression data are combined with other genetic, functional, phenotypic, and disease-oriented data. This allows GXD to provide tools for researchers to evaluate expression data in the larger context, search by a wide variety of biologically and biomedically relevant parameters, and discover new data connections to help in the design of new experiments. Thus, GXD can provide researchers with critical insights into the functions of genes and the molecular mechanisms of development, differentiation, and disease.

## Introduction

Recent technological advances have made it possible to rapidly determine the sequences of individual human genomes and to correlate genetic mutations with human diseases. Evolutionarily closely related to humans, the mouse is a pivotal model system for determining the molecular mechanisms that lead from specific mutations to developmental defects and disease phenotypes. In mouse, specific constitutive and conditional mutants can be easily generated, and tissues from many different strains and mutants, as well as all developmental stages, can be obtained for gene expression analyses. These expression data can then be correlated with phenotypic and disease data to gain insights into the function of genes and the molecular mechanisms that underlie human development, differentiation, and disease.

The objective of the Gene Expression Database (GXD) is to support and facilitate the studies of the molecular mechanisms that underlie developmental and disease processes. GXD systematically collects and integrates different types of expression data from wild-type and mutant mice through curation of the published literature and by collaboration with large-scale projects and makes them available to researchers in an extensive and easily searchable database (Finger et al. 2011; Smith et al. 2014a). Further, as an integral component of the larger Mouse Genome Informatics (MGI) resource, GXD combines its data with all the other genetic, genomic, function, phenotypic, and disease-related information in MGI, thus placing these expression data in context and making them readily accessible to many types of biologically and biomedically relevant database searches (Eppig et al. 2015; Smith et al. 2014b).

The importance of recording and integrating mouse expression data and placing them in a larger biological context cannot be overstated. It is impossible for any single individual to keep abreast of all the biomedical research data that are generated yearly, let alone to memorize all these data and their connections. The ability to find results of previous experiments quickly can save investigators months of research time, both in the library and in the laboratory. In addition, GXD and MGI enable researchers to discover new data connections, thus allowing them to develop scientific hypotheses and to design new experiments.

In the following paragraphs, we will discuss: the contents of GXD; how and why expression data are recorded in standardized ways; the integration of expression data with other data in MGI; and the tools provided by GXD to explore these data.

## GXD content

GXD collects endogenous gene expression information derived from wild-type and mutant mice. It includes data from all stages of development, including postnatal development, although the main emphasis is gene expression during the embryonic period. GXD provides researchers a comprehensive survey of the embryonic expression literature, detailed expression data, and tools to examine these data. Because different types of expression assays yield different information about gene products at the RNA and protein level, GXD has been designed as a system that can integrate multiple types of expression data (Ringwald et al. 1994). GXD’s emphasis has been, and continues to be, on data from RNA in situ, immunohistochemistry, knock-in reporter, RT-PCR, northern blot, and western blot experiments. Links to array and high-throughput sequencing expression data at NCBI GEO (Barrett et al. 2013) and the Expression Atlas at EMBL-EBI (Petryszak et al. 2014) are provided as well, and closer integration of these data within GXD is planned for the future.

GXD’s data content and acquisition efforts are unique, integrating heterogeneous expression data from disparate sources. GXD is the only database that systematically curates mouse developmental expression data from the literature. The GXD curators have read and entered the results from thousands of published papers into GXD. Additional data are acquired via electronic data submissions and through collaborations with large-scale data providers. The large-scale projects whose data are in GXD include: GenePaint (Visel et al. 2004), Eurexpress (Diez-Roux et al. 2011), the Brain Gene Expression Map (BGEM; Magdaleno et al. 2006), and the GenitoUrinary Development Molecular Anatomy Project (GUDMAP; Harding et al. 2011). Thus, the data in GXD represent the results of research performed by small- and large-scale laboratories worldwide. GXD currently contains detailed expression results from almost 70,000 experiments and data for nearly 1.5 million expression results examining the expression of approximately 13,900 genes. This includes data from more than 2100 mouse mutants. In addition, it has over 265,000 images of the original data, allowing researchers to view and interpret the experiments themselves. Eighty-two percent of the data are from RNA in situ hybridization studies and 10 % from RT-PCR experiments, reflecting the detailed spatial resolution and sensitivity required in developmental expression studies.

## Comprehensive survey of the embryonic expression literature

GXD provides researchers with an effective way to search the mouse embryonic expression literature. Curators survey journals to find all publications that contain studies of embryonic gene expression using the assay types that GXD collects. They then index these publications with regard to the genes that have been studied, the expression assay types used, and the ages analyzed. Significantly, the curators review the entire article, including supplemental data, and use standard nomenclature for the genes. These annotations are then combined with bibliographic information from PubMed to generate the Gene Expression Literature index.

**Fig. 1:**
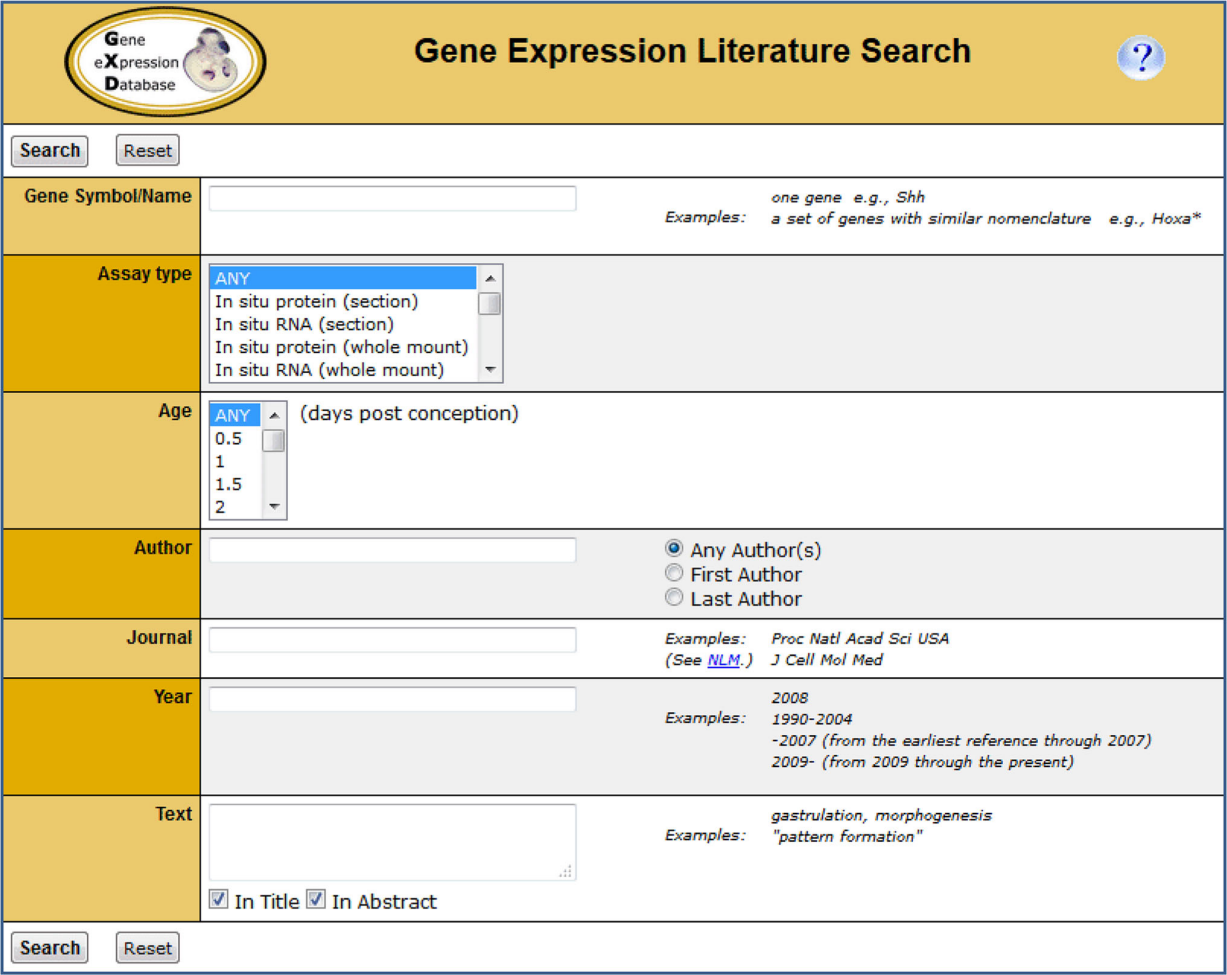
The Gene Expression Literature Search (http://www.informatics.jax.org/gxdlit) allows researchers to quickly find publications in the mouse embryonic expression literature. Users can search for genes and ages analyzed and expression assay types used, as well as by bibliographic information or specific words in the title or abstract

 Presently the Gene Expression Literature index covers over 23,000 references reporting expression data for more than 15,000 genes. It is complete and up-to-date from 1990 onward for all major journals. On average over 1100 papers are added to the index each year, demonstrating the impossibility for any individual to keep track of all the relevant literature. All of this information is available by means of the Gene Expression Literature Search (Fig. 1), allowing researchers to quickly find publications that report expression data of interest to them.

## Standardized, detailed expression data

**Fig. 2:**
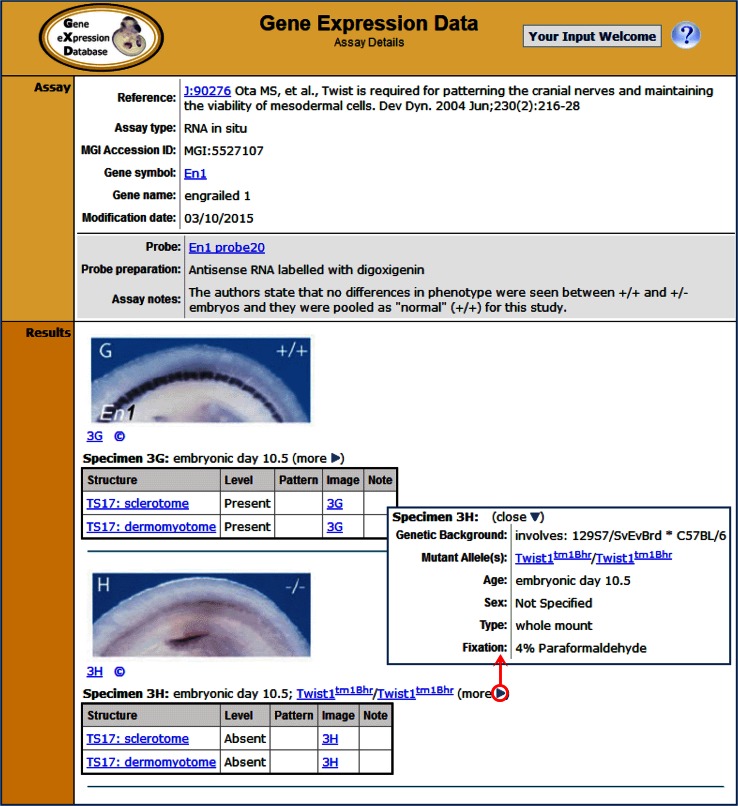
Assay Details pages reveal the detailed content of GXD’s expression records. A record for a RNA in situ experiment is shown. The Assay section reports the reference from which the data were derived, the assay type, the gene analyzed, and the probe used, with links to more details about each. The Results section reports the tissue (Theiler stage and anatomical structure) analyzed, as well as the level and pattern of expression. Images of the original expression data are displayed together with the corresponding annotations whenever possible. Major specimen details such as the age and mutant alleles are always displayed on this page; other information is accessible by means of the “more” toggle (*circled*)

GXD contains detailed records of experimental expression results derived from publications in the literature index and large-scale downloads. These records are created by the GXD curators. Figure 2 shows an example of a RNA in situ hybridization assay record. The assay records describe the results obtained for each specimen (or gel lane), including the presence (or absence) of expression for each anatomical structure examined, as well as details about the genetic background of the specimen, the molecular probe and the experimental conditions used. Images of the original expression data accompany the annotations whenever possible.

The GXD curators make extensive use of controlled vocabularies and ontologies when creating these expression records. These vocabularies can be simple lists, such as adjectives describing the pattern and level of gene expression, or large and highly structured, such as our anatomy ontology. Adding data to a database, such as GXD, involves more than collecting it from different sources. It must be reviewed and standardized, so it can be linked to and integrated with other data in the database. As will be discussed below, genes, alleles, and anatomical structures are among the key points of data integration in MGI, enabling the search capabilities that GXD provides.

To enable proper data integration and searching, it is important that GXD curators accurately identify the gene whose expression was analyzed, whether the data were obtained from the literature or from a large-scale download. Identifying the genes studied in a publication can be difficult because authors do not always use official nomenclature. Thus, a gene symbol given in a paper may refer to one of several possible genes. When entering data from the literature, curators use context, references cited in the paper, and probe or antibody information to accurately identify the genes examined. When reviewing data obtained from large-scale projects, probe information is essential for correct gene identification. The large-scale efforts usually generate probes for thousands of genes at the beginning of the project and then take several years to complete. During this time new genome assemblies may be released and new gene models created. When these data are submitted to GXD, the curators reanalyze all probe-to-gene associations to ensure that they are still correct. Because the MGI gene catalog is reviewed with each new genome assembly, once data are entered into GXD the correct probe-to-gene associations will be maintained and the expression information will remain associated with the correct genes.

GXD curators also ensure expression data from mutant mice are associated with the correct allele. Different alleles of the same gene may have quite different phenotypes. Therefore, in order to integrate expression and phenotype information, curators must accurately identify which mutant was used.

GXD annotates expression results using the Mouse Developmental Anatomy Ontology, developed in collaboration with the Edinburgh Mouse Atlas Project (Hayamizu et al. 2013). The ontology is extensive, containing more than 28,000 stage-specific anatomical terms hierarchically organized from tissue to tissue substructure. The detailed ontology and its hierarchical structure allows for the integration of data of varying levels of granularity and enables searches that include anatomical structures and their substructures. Within MGI this ontology is also being used to annotate sites of expression and activity data for recombinase alleles (the “Cre portal”; http://www.creportal.org), and its terms are used in the naming of Gene Ontology and Mammalian Phenotype ontology terms, laying the groundwork for closer integration with these data in the future.

 It is important to note that it is our curatorial policy to annotate expression patterns strictly based on what the authors say in the text of the manuscript and the figure legends. GXD curators do not interpret the published images. To do otherwise would be an error-prone process because the authors are experts in their fields and base their interpretations on more data than is provided in the publication. GXD annotations are associated with the images so that users can see the primary data and interpret it themselves. GXD images, together with annotations, are also being made available to EMAGE (Richardson et al. 2014) for spatial mapping, and GXD provides links from image entries to corresponding entries in EMAGE. (Due to the inherent variation of embryos, spatial mapping is only applicable to a subset of in situ data from wild-type mice.)

## Easy-to-use search tools

**Fig. 3:**
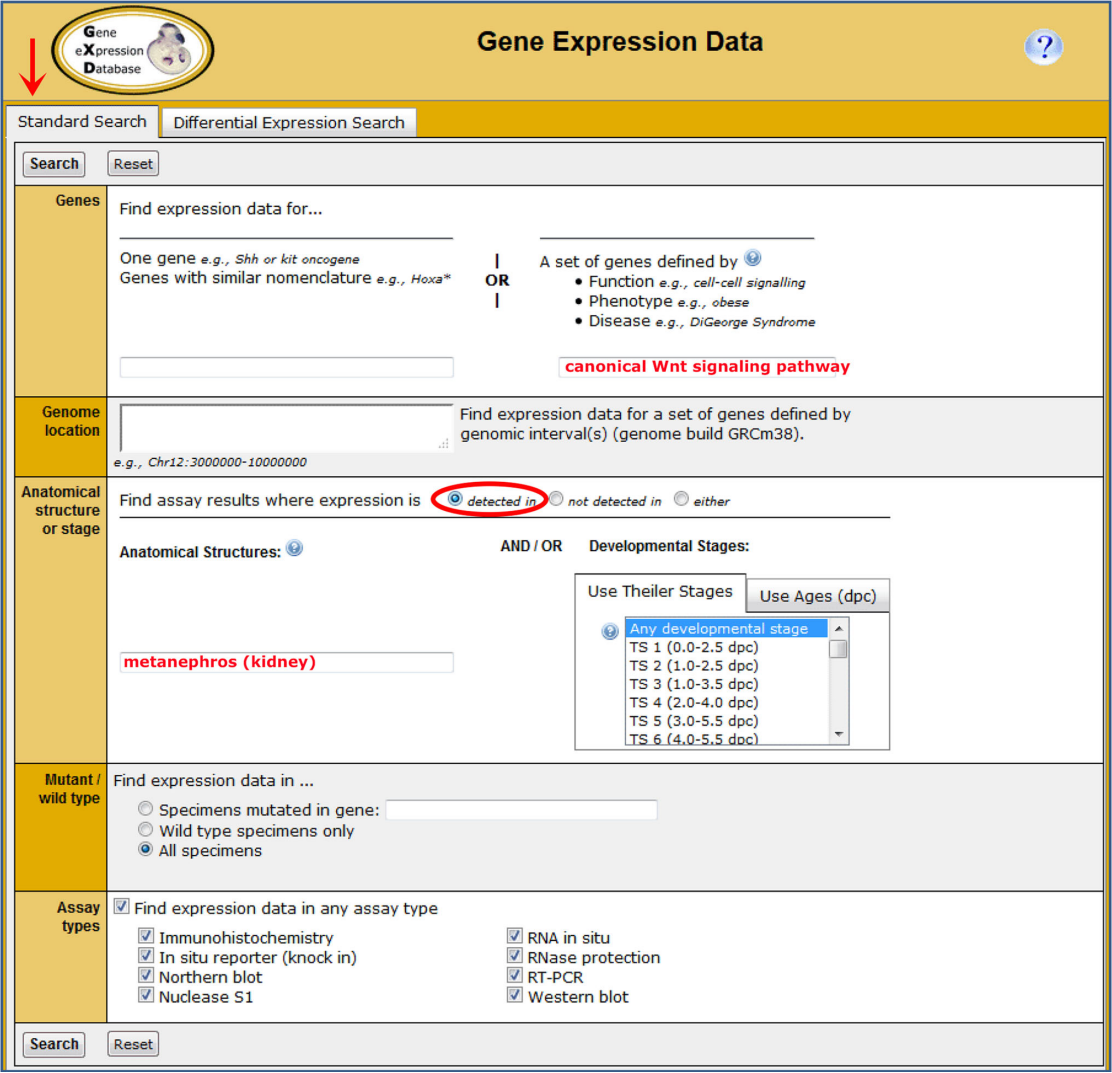
The Gene Expression Data Query Form (http://www.informatics.jax.org/gxd) features two search tabs: Standard and Differential Expression. The Standard Search (above) provides the most flexibility, allowing querying of expression data using the parameters of most interest. The Genes section allows searching by a specific gene or for a set of genes based on their function [as defined by Gene Ontology terms (Gene Ontology Consortium 2015)]; their association with mouse phenotypes [as defined by Mammalian Phenotype Ontology terms (Smith and Eppig 2012)]; or their association with human diseases [as defined by Online Mendelian Inheritance in Man (OMIM) terms (Amberger et al. 2015)]. The Genome location section makes it possible to limit expression searches to genes located in a particular chromosomal region, which is useful when hunting for candidate genes. The Anatomical structure or stage section allows searching for expression data in specific anatomical structures and/or developmental (Theiler) stages or ages. Anatomical searches take advantage of the hierarchy of the anatomy ontology, returning annotations to substructures as well as to the structure itself. The Mutant/wild-type section can be used to limit the searches to expression data from wild-type mice or from specific mutants. The Assay types section allows for the selection of desired expression data types. Auto-fill functionality helps to find appropriate search terms. The illustrated search is “Which canonical Wnt signaling genes have been studied in the kidney?” Figure 5 shows the corresponding search results pages

**Fig. 4:**
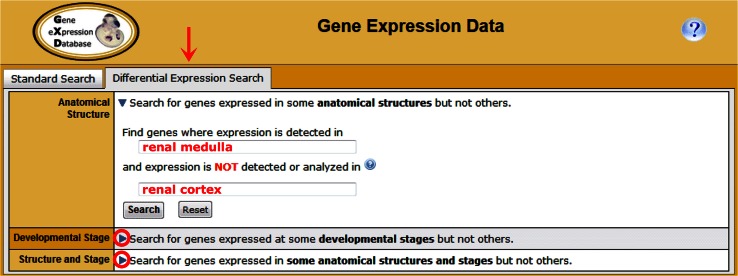
The Differential Expression Search on the Gene Expression Query Form allows searching for genes that are expressed in some anatomical structures but not others and/or at some developmental stages but not others. To facilitate these searches, the form has been divided into three sections; each is optimized for entry of different combinations of anatomical structures and/or developmental stages. One section is expanded in the illustration; the other two may be expanded using the toggles (*circles*). The illustrated search asks, “Which genes are expressed in renal medulla but not renal cortex?” Figure 7 shows part of those search results

Users can search the detailed expression data in GXD using the Gene Expression Data Query Form. It has two search tabs: Standard and Differential Expression. The Standard Search (Fig. 3) provides the most versatile means to query for expression data. It has a variety of fields, enabling searches using one or many parameters. Users can search by the details of the expression patterns they want and/or the attributes of the genes of interest. Due to the hierarchical organization of the anatomy ontology, searches for gene expression in an anatomical structure, such as kidney, will also return annotations to its substructures, such as renal cortex and medulla. Thus, both broad questions, such as “Where is *Pax6* expressed?”, or complex questions, such as “Which canonical Wnt signaling pathway genes are expressed in the kidney?”, can be asked. The Differential Expression Search (Fig. 4) allows searching for genes that are expressed in some anatomical structures but not others and/or in some developmental stages but not others. Thus, one can ask, “What genes are expressed in the renal medulla but not the renal cortex?” These are just a few examples of the many searches that are possible when using the Gene Expression Data Query Form.

## Search returns that allow for data refinement and exploration

**Fig. 5:**
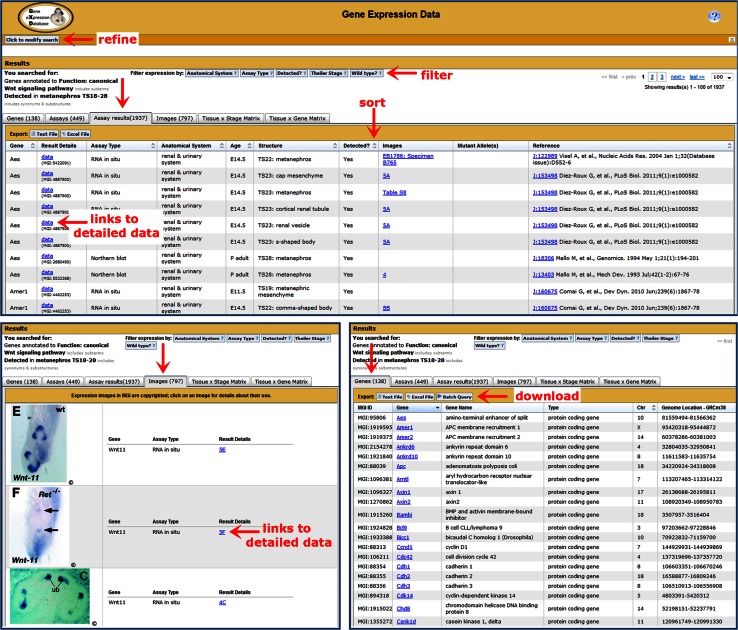
Gene Expression Data searches return summaries for the expression results that match the search parameters, organized under six separate tabs. Each summary presents the data with a different level of detail and focus. The number of matching records is indicated in the tab header. The Assay results tab (*upper*) is displayed by default. It lists the gene studied, the assay type used, the anatomical system, age and tissue examined, indicates whether expression was detected, provides a link to the corresponding images, lists the mutant alleles of the specimen (if applicable), and provides the reference from which the data were derived. Links in the Result Details and Images columns lead to detailed expression records, such as the one shown in Fig. 2. *Arrowheads* in column headers indicate that the column is sortable (one set is indicated). The Assay results tab (as well as the Genes tab) allows for export of results in text and spread sheet formats (buttons in table header). The Images tab (*lower left*) shows all the images that match the search criteria, together with the gene(s) examined in that image and the assay type used and provides a link to the corresponding part of the detailed expression record. The Genes tab (*lower right*) provides a list of the genes that match the query. The export feature can forward the genes list to either the MGI batch query or MouseMine, thereby allowing searches for additional information, such as the function, phenotype and/or disease terms associated with the genes of interest. Expression summaries can be refined using the “Click to modify search” button or using the filter options provided on the summary page. The content of all of the summaries will change accordingly. To filter by anatomical structures or by gene, use the row and column filters available on the Tissue × Stage Matrix (Fig. 6) or Tissue × Gene Matrix (Fig. 7) respectively

**Fig. 6:**
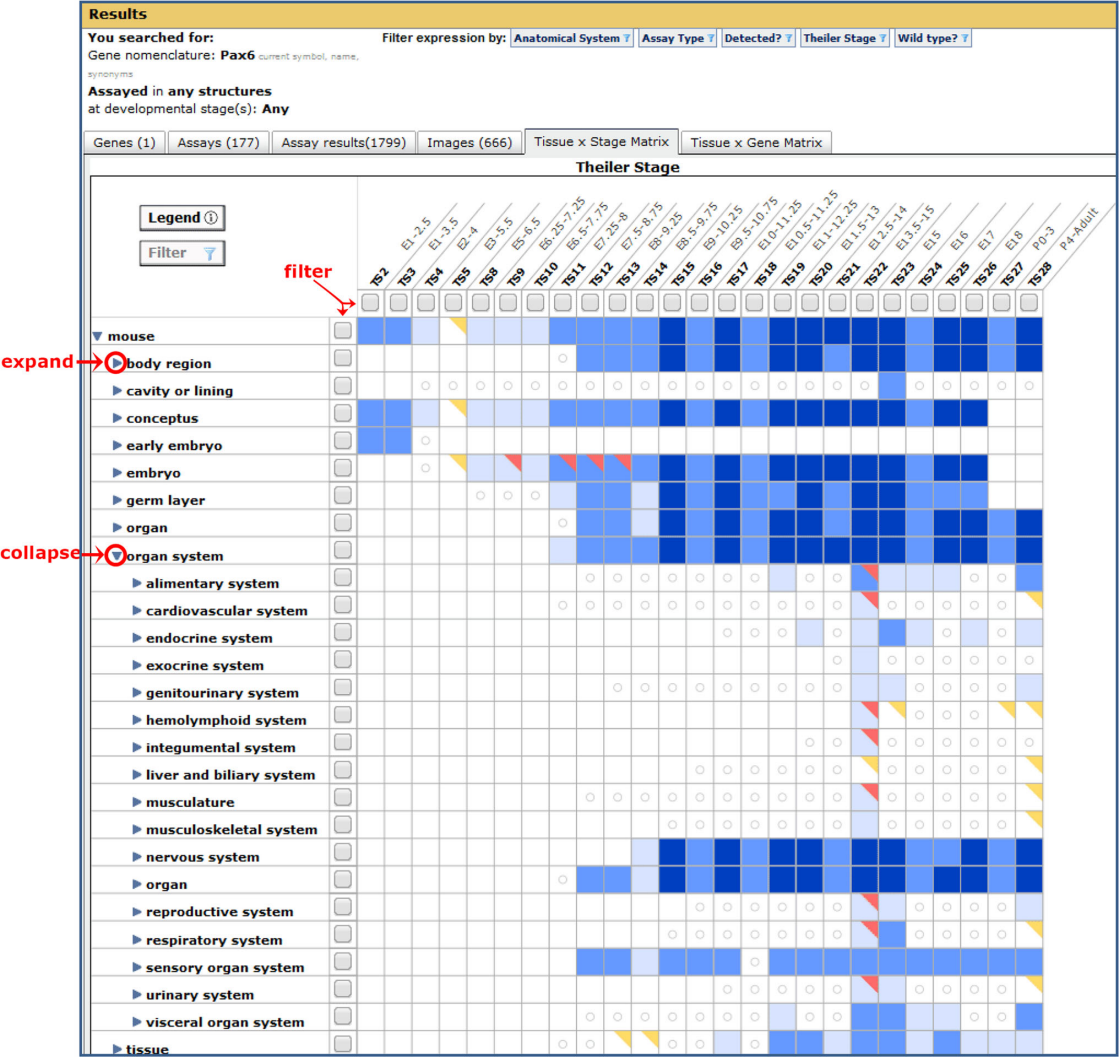
The Tissue × Stage Matrix on GXD search summaries provides a spatial and temporal overview of search results. The matrix columns show the Theiler stages where expression was examined, and the rows display the tissues that were examined. Clicking on the toggles (*circled*) expands (or collapses) the anatomy hierarchy. Results can be refined by tissue and developmental stage using the *gray* check boxes (*arrows*) to filter the search results. The *colors* in the matrix cells indicate the type of annotations: *blue* for presence of expression and *red* for absence of expression. The cell colors get progressively darker when there are more supporting annotations; an online legend details the number of annotations represented by each *color*. Cells with a *red* corner indicate that expression has been reported as being both present and absent in that tissue. Although this may be because different laboratories made conflicting observations, it is more likely to be due to other factors such as: differences in the genotype or sex of the specimen; sensitivity of the assay used; or a difference in the transcript variant assayed. A cell with a *gold* corner indicates that there are expression results for substructures of the tissue that were reported as being either absent or ambiguous. A *circle* in a cell indicates the tissue exists at that developmental stage but GXD has no expression data for it, whereas a blank cell indicates that the tissue does not exist at that stage. To access the data for specific tissue by stage combinations, click the cell to open a pop-up. It displays a table with more details about the annotations and has buttons which, by acting as a filter, will allow viewing of those annotations in either the Assay results or Images tab (Fig. 5)

**Fig. 7:**
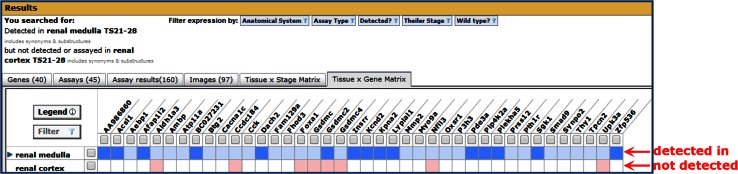
The Tissue × Gene Matrix tab on GXD search summaries enables the comparison of expression patterns of different genes. The matrix columns show genes whose expression was examined, and the rows display the tissues that were examined. By using the *gray* check boxes to filter the search results, the matrix also allows for refinement of search results by gene, as well as tissue. Most of the cell coloring conventions are the same as those for the Tissue × Stage Matrix, as described in Fig. 6, with the exception that in this matrix the white cells indicate there are no annotations in the database. The Tissue × Gene Matrix is especially useful when interpreting the return for Differential Expression searches (Fig. 4). This figure shows the matrix generated by a search for genes expressed in the renal medulla but not detected or assayed in renal cortex. The matrix makes it immediately clear which genes have been examined and shown not to be expressed in the renal cortex (*red cells*) and those for which there is no expression information about the renal cortex in GXD (*white cells*), either because these results have not been reported in the literature or because they have not yet been recorded in GXD

The GXD searches return pages with six tabbed summaries (Figs. 5, 6 and 7). Each summary has a different focus, allowing researchers to more easily find the data they are interested in and view it at the desired level of detail. This facilitates the understanding and handling of search results.

The Assay Results tab (Fig. 5) provides the most detailed summary. It lists the structures assayed and whether or not expression was detected, as well as the gene analyzed, assay type used, information about mutant genotypes, reference information, links to associated images, as well as links to the detailed pages discussed above. It also has export features, allowing results to be easily downloaded in text or spreadsheet format.

The Images tab (Fig. 5) allows for review of expression images that match the search criteria. This search return is made possible by the GXD curators’ annotation of the metadata associated with these images. Images are displayed in GXD when we have permission from the publisher or when contributed by a data provider.

The Genes tab (Fig. 5) provides a list of the genes that match the search criteria. The list can be downloaded in text or spreadsheet format or forwarded to either the MGI batch query (Bult et al. 2010) or MouseMine (http://www.mousemine.org). This allows searching for additional information associated with the genes of interest, such as function, phenotype, and/or disease annotations.

There are two matrix view summaries: Tissue × Stage and Tissue × Gene. By default these matrices provide a high-level overview. However, toggles can be used to expand (and collapse) the matrices along the tissue axis to reveal greater levels of detail in the areas of particular interest. The number of returned results can be refined using filters.

The Tissue × Stage Matrix provides a summary of the temporal and spatial expression patterns of genes. This is a good way to get an overview of the expression data for a single gene, as illustrated in Fig. 6. Alternatively, using the Gene Expression Data Matrix link on the GXD home page (http://www.informatics.jax.org/expression.shtml) generates a Tissue × Stage Matrix that displays all of GXD’s data. Then, using the toggles and filters, users can view and iteratively select expression data for the specific tissues and/or developmental stages of interest.

The Tissue × Gene Matrix is useful when comparing expression patterns between genes. This can be especially helpful when using the Differential Expression search (Fig. 4) to find genes expressed in one structure and not another. Figure 7 shows a matrix generated by such a search. Each column corresponds to a gene whose expression pattern matched the search criteria. The row for the structure whose expression was searched for, renal medulla in this case, is filled with blue cells; the intensity of the color indicates the number of annotations in the database. The row for the other structure (renal cortex) is filled with red or blank cells. Red cells indicate expression of that gene was shown to be absent in the structure; blank cells indicate the expression of that gene has either not been analyzed or has not been recorded in GXD for the tissue.

## Access to GXD

GXD is available through the MGI web site, http://www.informatics.jax.org. The GXD home page can be accessed directly at http://www.informatics.jax.org/expression.shtml; by means of the MGI home page by following the topic “Gene Expression Database (GXD)”; or from any other page within MGI by clicking the “Expression” tab of the navigation bar. This page gives access to GXD’s search forms and provides more information about GXD, including FAQs, help documents, news announcements, and links to other mouse expression resources.

GXD welcomes direct data submission. The creation of detailed expression records is time consuming. Hence, not every paper in the literature index has detailed expression records. Data submission is the most effective way to ensure that your data will be included in GXD. Inclusion of your data in GXD allows for its integration with the other data in GXD and the larger MGI resource. This will make your data available for database searching, thus adding to its utility and increasing its exposure to the scientific community. Furthermore, GXD ensures that data and data connections are maintained and kept up-to-date even if the gene models or the gene names used in the literature have changed. You can find instructions for data submission on the “Send us your data” tab at the bottom of the GXD Home Page (http://www.informatics.jax.org/mgihome/GXD/GEN/gxd_submission_guidelines.shtml).

GXD and MGI have dedicated User Support personnel. They can be contacted by emailing mgi-help@jax.org or via the “Contact Us” link in the navigation bar of our web pages. User Support and GXD curators actively seek to provide presentations, demonstrations, and training sessions on GXD and the larger MGI resource at many meetings. User Support can also provide on-site training workshops, as well as remote interactive sessions, upon request. GXD is continually looking for ways to improve the user experience. Direct user interactions, surveys, and collaborations provide valuable feedback on the project from those who use our data. Your feedback is used to prioritize database improvements.

## Conclusions

The first full version of the GXD database went online in July 1998 with 3600 publications in the literature index and 32,000 detailed expression results analyzing 517 genes (Ringwald et al. 1999). Since then the usefulness of GXD has grown tremendously as the data content has increased and more powerful web tools have been developed. Going forward we will continue these efforts to integrate gene expression data in the proper biological and analytical context by adding new types of expression data and developing even more advanced search and display tools. Thus, GXD will continue to be an essential resource for researchers who study the molecular basis of development, differentiation, and disease.
